# Autoimmune thyroid disease modifies the clinical expression of hand osteoarthritis in older people: a third National Health and nutrition examination survey study

**DOI:** 10.3389/fmed.2024.1445188

**Published:** 2024-08-20

**Authors:** Clement E. Tagoe, Wanyi Wang, Helena H. Kwon

**Affiliations:** ^1^Division of Rheumatology, Department of Medicine, Albert Einstein College of Medicine, Bronx, NY, United States; ^2^Elite Research, LLC, Irving, TX, United States; ^3^Albert Einstein College of Medicine, Bronx, NY, United States

**Keywords:** autoimmune thyroid disease, Hashimoto’s thyroiditis, hand osteoarthritis, Heberden’s nodes, Bouchard’s nodes, hand pain

## Abstract

**Introduction:**

The risk factors linked to hand osteoarthritis (OA) that contribute to its distinct symptoms and clinical presentation are not thoroughly understood. This study aimed to examine whether the autoimmune thyroid disease (AITD) autoantibodies, anti-thyroid peroxidase antibody (TPOAb), and anti-thyroglobulin antibody (TgAb), associate with hand OA and symptomatic hand OA in the Third National Health and Nutrition Examination Survey (NHANES III).

**Materials and methods:**

We included 2,429 persons from NHANES III ≥60 years of age. Data on hand OA or symptomatic hand OA were examined with respect to their associations with TPOAb and TgAb. Log-binomial and modified Poisson regression models were fit to examine the associations between the anti-thyroid autoantibodies and hand OA or symptomatic hand OA.

**Results:**

Higher levels of TPOAb were associated with a higher prevalence of symptomatic hand OA in the unadjusted (*PR* = 1.182, *p* = 0.024) and adjusted models after controlling for age, gender, and diabetes (*PR* = 1.174, *p* = 0.039). This association was no longer significant when positive TPOAb was considered a categorical variable with four levels and compared with negative TPOAb. TgAb showed a trend toward being positively associated with symptomatic hand OA (*p* < 0.10). When positive TgAb was considered a categorical variable with four levels and compared with negative TgAb, the highest quartile was associated with a higher prevalence of symptomatic hand OA than negative TgAb in the unadjusted (*PR* = 2.242, *p* = 0.008) and adjusted models (*PR* = 2.045, *p* = 0.038). There was no significant association between TPOAb or TgAb and hand OA.

**Conclusion:**

Higher levels of TPOAb may be associated with the presence of symptomatic hand OA in persons ≥60 years old. Persons ≥60 years old with the highest quartile levels of TgAb may be more likely to present with symptomatic hand OA.

## Introduction

Osteoarthritis (OA) of the hand is associated with the presence of bony deformities including Heberden’s nodes, Bouchard’s nodes, and deformity of the first carpometacarpal (CMC) joint, with or without angular deformity (1). The factors conferring risk and determining the specific joint distributions in hand OA are not well understood. Unlike knee OA, where body mass index (BMI) plays a pivotal role in disease causation, the influence of gravitational forces is less likely to be critical in initiating cartilage destruction in hand OA (2). Indeed, population studies did not show a consistent association of BMI with the presence of hand OA across gender groups (3). The evidence from epidemiologic studies demonstrated that older age, female gender, prior trauma or heavy manual labor, and family history are the greatest risk factors for having hand OA (4, 5). A recent review suggested a role in diabetes but limited evidence was noted (6). Better knowledge of the risk factors associated with hand OA is needed to understand its specific symptoms, including pain and stiffness, joint patterns, gender, and age prevalence.

Although OA has historically been described as a degenerative joint disease, recent evidence suggests the possibility of inflammatory and immunological processes affecting the entire joint as an organ (7). AITD is a female-predominant autoimmune inflammatory thyroiditis typified by lymphocytic infiltration of the thyroid gland with multiple clinical sequelae ranging from asymptomatic disease in the majority of cases to hyperthyroidism and hypothyroidism (8). In epidemiological research, the condition is identified through the prevalence of anti-thyroid autoantibodies (ATAs) found in most affected individuals (9). Chronic lymphocytic thyroiditis and its goitrous form, Hashimoto’s thyroiditis, are forms of AITD more closely associated with several musculoskeletal syndromes than Graves’ disease, including OA in the hands and knees, OA in the spine, chronic widespread pain, and fibromyalgia syndrome (10, 11).

This study investigated for the first time the possible association of AITD, as defined by the presence of the ATA anti-TPOAb and anti-TgAb, with clinical hand OA in a large population-based cohort. Given similarities in the epidemiology of the two conditions, including the female predominance and age prevalence in the middle-aged and the elderly, we hypothesized an association between the presence of thyroid autoantibodies and hand OA (9). This would support an immunological role, at least in part, for the etiology of hand OA. We sourced the Third NHANES III Study because clinical data were available for hand OA, including the presence of chronic hand pain/stiffness, the bony deformities of Heberden’s nodes, Bouchard’s nodes, and the first CMC joint deformities, as well as for the ATA TPOAb (also referred to as the antimicrosomal antibody in NHANES III) and TgAb, in a large cross-sectional sample representative of the United States population.

## Materials and methods

### Data source

The NHANES III was the last non-continuous NHANES study completed by the United States National Center for Health Statistics (NCHS) and was conducted in Phase 1 (1988–1991) and Phase 2 (1991–1994) using complex, multi-stage, stratified, clustered national probability samples of civilian, non-institutionalized persons in the US population. Children aged 2 months– 5 years, persons ≥60 years, Black non-Hispanics, and Mexican Americans were oversampled to improve the precision of estimates in those groups. The planning and execution of the study involved close cooperation and collaboration with the communities studied. The community engagement, consent process, operation, and procedures for NHANES III have been described in detail (12). Participant consent for publication was not required for the local conduct of this study, and the data source exists in the public domain at https://wwwn.cdc.gov/nchs/nhanes/nhanes3/default.aspx. The analysis for the study was approved by the Institutional Review Board (IRB) of the Albert Einstein College of Medicine, NY, and followed the recommendations of the Strengthening the Reporting of Observational Studies in Epidemiology (STROBE) statement checklist for cross-sectional studies (13) (STROBE Checklist - Supplementary Table S1).

### Study population

**Figure 1:**
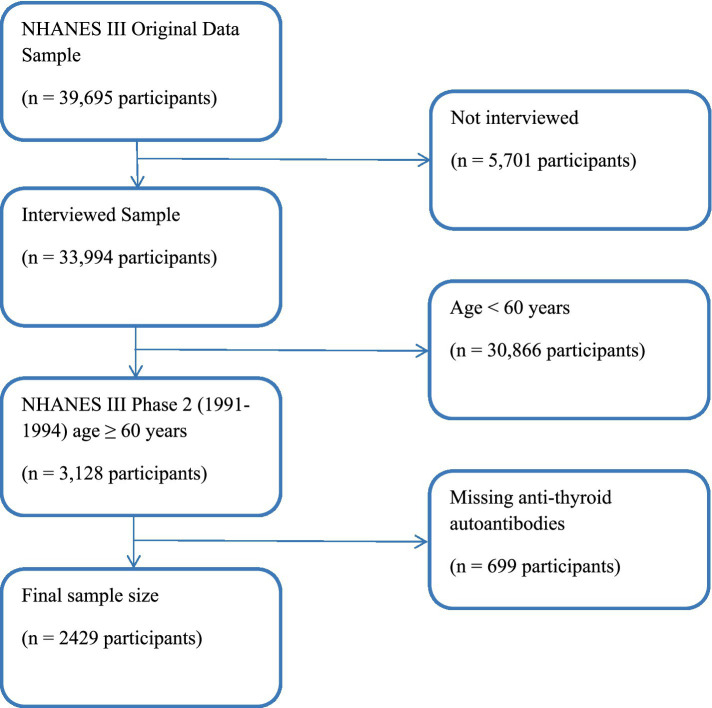
Flow diagram of study participant selection process.

A sample of 33,994 NHANES III participants was interviewed; of which, 3,128 were aged 60 years and over and were in Phase 2 of the study, where data for OA were collected. A total of 2,429 participants ≥60 years old had complete data on TPOAb and TgAb (Figure 1). The home interviews were followed within a few weeks by mobile examination center (MEC) visits, which included physician performed physical examinations and the acquisition of knee and hand radiographs. Although the knee radiographs have been read and analyzed, the hand films have not. The classification criteria adopted by the American College of Rheumatology (ACR) for hand OA did not require radiographs (1). Thus, clinical data for hand OA were available as part of the home interview and MEC examination.

### Outcome measures

Bony deformities were assessed as clinical markers of the presence of hand OA and included the following: Heberden’s nodes—present at the distal interphalangeal (DIP) joints, Bouchard’s nodes—present at the proximal interphalangeal (PIP) joints, and first CMC joint deformity—present at the first CMC joints. Chronic hand pain was recorded as the presence of a “yes” response to the question, “Have you ever had pain in your hands on most days for at least 6 weeks? This also includes aching and stiffness.” Clinical hand OA was defined using the ACR Classification Criteria modified for the NHANES III dataset and required the presence of hard tissue enlargement of 2 or more of the 10 selected joints (the second and third DIP, second and third PIP, and the first CMC joints of both hands), fewer than three swollen metacarpophalangeal (MCP) joints, and either hard tissue enlargement of 2 or more DIP joints at any site, or deformity of 1 or more first CMC joints (1). The criteria were modified from the NHANES III symptomatic hand OA study of Dillon et al. (14). Our modification emphasized the classification tree structure in the ACR classification criteria and the logic table for the NHANES III classification is summarized in Table 1 (1). The presence of wrist swelling, the presence of morning stiffness exceeding 60 min, the presence of positive rheumatoid factor, the presence of at least three swollen MCP joints, wrist or MCP swelling in bilateral hands, and the presence of rheumatoid nodules were criteria used to exclude individuals with rheumatoid arthritis (15) (Table 1). A history of chronic hand pain, aching, or stiffness for at least 6 weeks was identified as subjects with symptomatic hand OA.

**Table 1:** Summary of classification criteria for hand osteoarthritis and symptomatic hand osteoarthritis.

Step	Criteria	*n*	Weighted %
1	Bilateral Heberden’s nodes	1006	46.2
2	Identify first CMC deformity, Bouchard’s nodes, and Heberden’s nodes		
	2.1. Deformity of 1 or more first CMC joints	412	18.4
	2.2. 1 or more Bouchard’s nodes	675	29.8
	2.3. 1 Heberden’s node	272	12.0
3	Combination of CMC deformity with Bouchard’s nodes and Heberden’s nodes or with Heberden’s nodes only from Step 2	42	1.6
4	Sum of Steps 1 and 3	1048	46.7
5	Rheumatoid arthritis criteria		
	5.1. RA criteria 1 - Morning stiffness over 1 h	34	1.4
	5.2. Subcutaneous nodules on the shaft of forearm	7	0.3
	5.3. Positive rheumatoid factor	64	2.6
	5.4. Wrist swelling	8	0.3
	5.5. Presence of at least 3 swollen MCP joints	7	0.3
	5.6. Wrist or MCP swelling with bilateral hands	4	0.2
6	Identify rheumatoid arthritis		
	6.1 Meet three or more criteria from Step 5	2	0.1
7	Identify Hand Osteoarthritis (Subtract 6.1 from 4)	1046	46.6
8	Identify Symptomatic Hand Osteoarthritis out of Step 7 (Hand pain, aching, or stiffness, on most days for at least 6 weeks)	212	8.6

Overall *N* = 2429.

### Demographic information

Participants ≥60 years were included in this study. Ethnicity was classified as non-Hispanic White, non-Hispanic Black, Mexican American, and others (defined as other Hispanics, Asians, and Native Americans in NHANES III). Education was stratified as less than or equal to 12 years and greater than 12 years. BMI was divided into underweight/normal weight (BMI < 25 kg/m^2^), overweight (25 ≤ BMI < 30 kg/m^2^), and obese weight status (BMI ≥ 30 kg/m^2^). The occupation was grouped as manual workers and non-manual workers, as previously described by Dillon et al. (2). Current smoking was captured by the responses “yes” to the questions “Do you smoke cigarettes now?” and “Have you smoked at least 100 cigarettes during your entire life?.” Former smoking was recorded as the response “yes” to the latter and “no” to the former question, respectively. Never smokers responded no to either question.

### Clinical variables

The NHANES III laboratory methods on clinical variables have been published in detail elsewhere (16). Abnormal values of TPOAb and/or TgAb, where the normal range was <0.5 IU/mL and < 1.0 IU/mL, respectively, denoted the presence of AITD (16). Serum C-reactive protein (CRP) was measured with a range of 0.3–25.2 mg/dL. The methodology for the determination of serum thyroid-stimulating hormone (TSH) and thyroxine (T4) has been described in a previous publication (17). Euthyroidism, hyperthyroidism, and hypothyroidism were defined serologically as corresponding to TSH levels of 0.4–4.6 μIU/ml, < 0.4 μIU/ml, and > 4.6 μIU/ml, respectively. Participant-reported diabetes was determined by a “yes” to the question “Have you ever been told by a doctor that you have diabetes or sugar diabetes?.” Other medical conditions such as thyroid disease, goiter, and gout were self-reported by the participants during home interviews or MEC portions of the study.

### Statistical methods

Before the primary analysis, bivariate relationships were assessed between independent/dependent variables and the demographic/clinical variables to see whether any covariates should be controlled for. Rao–Scott chi-squared tests were used to compare the two categorical variables. Correlation analyses were performed between two continuous variables. Independent t-tests and one-way analysis of variances (ANOVAs) were used to examine the differences between continuous variables and categorical variables. Approximately 0.78% were missing information in the dataset. Because the proportion of the total missing data was less than 5% and the missing values were completely at random [little’s test of missing completely at random (MCAR): *χ*^2^ = 26.2, *p* = 0.999], pairwise deletion was used in the analysis. In the primary analyses, sample demographic and clinical characteristics were summarized with means and standard deviation (SD) for continuous variables, and weighted percentages and standard errors (SEs) for categorical variables. Log-binomial regression models were fit to predict hand OA from TPOAb and TgAb by both controlling and not controlling for some covariates. Due to the low percentages of symptomatic hand OA (< 10%), modified Poisson regression models with robust error variance were conducted to predict the outcome from TPOAb and TgAb. The adjusted prevalence ratio (PR) and 95% confidence interval (CI) were estimated by comparing each category to the reference group, which was adjusted for other covariates. Covariates were selected based on the bivariate analysis results and previously published findings (2). Continuous variables were normalized before being added into the models due to the various scales between the variables, thus the PRs for the continuous variables were interpreted as the disease incidence per SD change of the predictors. All data were analyzed using SAS version 9.4 (SAS Institute Inc., Cary, NC, United States) survey procedures taking into account the cluster, strata, and sampling weights. A two-sided *p*-value of <0.05 was considered statistically significant for all statistical tests.

## Results

### Sample characteristics

Among the 2,429 participants included in this study, over half of the sample were female (57.4%), with a mean age of 70.75 years. Most participants were non-Hispanic White (83.0%) and had education equal to or less than 12 years of duration (71.3%). A majority of participants did non-manual work (53.2%), and the average BMI was 27.15 kg/m^2^ (SD = 5.12). With respect to the clinical characteristics, most participants were euthyroid (85.7%), did not have thyroid disease (89.5%), or diabetes (88.0%), did not have goiter (97.4%), and had no gout (93.4%). However, more than half of the sample were smokers (53.0%). More details regarding the demographic and clinical characteristics are displayed in Table 2. Approximately half of the participants had hand OA (46.6%) but only 8.6% of the sample had symptomatic hand OA. TPOAb values ranged from 0.30 to 1000.00 IU/mL with a mean of 10.71 IU/mL, and TgAb values ranged from 0.70 to 3000.00 IU/mL with a mean of 9.52 IU/mL. Both ATAs were then dichotomized into negative and positive levels. As seen in Table 3, approximately 20.3% of the participants had positive TPOAb, and 17.6% of the participants had positive TgAb. When analyzing their relationships with hand OA, the positive values of both autoantibodies were further stratified into quartiles.

**Table 2:** Descriptive statistics for sample characteristics.

Categorical Variables	*n*	*Weighted%*	*SE of weighted %*
Demographics/Covariates			
Age (years)			
60–69	1084	49.5	2.2
70–79	804	35.0	1.6
80 and above	541	15.5	1.7
Sex			
Male	1137	42.6	1.3
Female	1292	57.4	1.3
Race/ethnicity			
Non-Hispanic White	1371	83.0	2.0
Non-Hispanic Black	454	7.7	1.0
Mexican American	501	2.4	0.3
Others	103	6.8	1.5
Education (years)			
Less than or equal to 12	1918	71.3	2.5
Greater than 12	497	28.7	2.5
BMI (kg/m^2^)			
Underweight/Normal	809	34.6	2.0
Overweight	997	40.4	1.5
Obese status	617	25.0	1.0
Occupation			
Manual	1374	46.8	2.4
Non-manual	1055	53.2	2.4
Smoking			
Yes	1249	53.0	1.6
No	1180	47.0	1.6
TSH levels			
Hyperthyroidism (<0.40mIU/L)	105	4.3	0.5
Euthyroidism (0.40–4.6 mIU/L)	2081	85.7	0.9
Hypothyroidism (>4.6 mIU/L)	243	10.0	0.7
Thyroid disease			
Yes	208	10.5	0.8
No	2219	89.5	0.8
Diabetes			
Yes	385	12.0	0.9
No	2042	88.0	0.9
Gout			
Yes	143	6.6	0.9
No	2286	93.4	0.9
Goiter			
Yes	56	2.6	0.4
No	2372	97.4	0.4
Continuous variables	*N*	Mean	SD
T4 (μg/dL)	2429	8.58	2.27
RF (IU/mL)	2423	46.85	354.94
CRP (mg/dL)	2426	0.56	0.92
Age (years)	2425	70.75	7.58
BMI (kg/m^2^)	2415	27.15	5.12

BMI, body mass index; TSH, thyroid-stimulating hormone; T4, thyroxine; RF, serum rheumatoid factor; CRP, C-reactive protein; *n*, sample size; SE, standard error; SD, standard deviation.

**Table 3:** Descriptive statistics for independent and dependent variables.

Categorical variables	*n*	Weighted%	SE of weighted %
Outcomes			
Hand OA			
Yes	1046	46.6	3.8
No	1383	53.4	3.8
Symptomatic hand OA			
Yes	212	8.6	0.8
No	2217	91.4	0.8
Independent variables			
TPOAb			
Negative	1975	79.7	1.3
Positive	454	20.3	1.3
TPOAb			
Negative	1975	79.7	1.3
0–25%	112	5.6	0.4
25–50%	115	5.0	0.7
50–75%	112	4.5	0.5
75–100%	115	5.2	0.6
TgAb			
Negative	2065	82.4	0.8
Positive	364	17.6	0.8
TgAb			
Negative	2065	82.4	0.8
0–25%	92	4.8	0.6
25–50%	93	4.7	0.6
50–75%	88	4.3	0.6
75–100%	91	3.8	0.3
Continuous variables	*n*	Mean	SD
TPOAb	2429	10.71	46.08
TgAb	2429	9.52	88.98

Hand OA, hand osteoarthritis; symptomatic hand OA, symptomatic hand osteoarthritis; TPOAb, anti-thyroid peroxidase antibody; TgAb, anti-thyroglobulin antibody; *n*, sample size; SE, standard error; SD, standard deviation.

### Associations between anti-thyroid peroxidase antibody and hand OA

To examine the relationships between TPOAb and hand OA/symptomatic hand OA, TPOAb was first treated as a continuous variable. When no covariates were controlled for, there was no significant relationship between TPOAb and hand OA (*p* = 0.934) (Table 4, model 1). When adjusting for age, gender, and diabetes, TPOAb was also not significantly related to hand OA (*p* = 0.999) (Table 4, model 2). The covariates were controlled for in the log-binomial model because they were significantly related to hand OA and/or TPOAb when analyzing the bivariate relationships in the preliminary analyses or reported in previous studies (2). The results indicate that females were more likely to be diagnosed as having hand OA than males, *PR* = 1.253 (95% CI: 1.12, 1.40), *p* < 0.001. Older participants were more likely to have hand OA, *PR* = 1.558 (95% CI: 1.27, 1.91), *p* < 0.001.

**Table 4:** Associations between TPOAb and hand OA/symptomatic hand OA.

	Hand OA						Symptomatic hand OA					
					95% CI of PR						95% CI of PR	
Models	*β*	SE	PR	*p*	LL	UL	*β*	SE	PR	*p*	LL	UL
Model 1												
TPOAb	0.004	0.05	1.004	0.934	0.91	1.11	0.167	0.07	1.182	**0.024**	1.02	1.37
Model 2												
TPOAb	−0.001	0.05	0.999	0.999	0.91	1.10	0.160	0.08	1.174	**0.039**	1.01	1.37
Age	0.444	0.10	1.558	**<0.001**	1.27	1.91	0.518	0.28	1.678	0.068	0.98	2.88
Female^a^	0.226	0.06	1.253	**<0.001**	1.12	1.40	0.304	0.19	1.356	0.242	0.94	1.95
Diabetes (Yes)^b^	−0.062	0.08	0.940	0.468	0.80	1.11	0.107	0.24	1.113	0.654	0.70	1.78
Model 3												
Positive TPOAb (Q1)^c^	0.095	0.11	1.100	0.371	0.89	1.35	0.056	0.42	1.057	0.895	0.46	2.42
Positive TPOAb (Q2)^c^	0.106	0.12	1.112	0.375	0.88	1.41	0.270	0.29	1.310	0.356	0.74	2.32
Positive TPOAb (Q3)^c^	0.005	0.16	1.005	0.975	0.73	1.38	0.297	0.36	1.345	0.416	0.66	2.75
Positive TPOAb (Q4)^c^	0.060	0.15	1.062	0.681	0.80	1.41	0.423	0.44	1.527	0.338	0.64	3.63
Model 4												
Positive TPOAb (Q1)^c^	0.033	0.10	1.033	0.999	0.84	1.26	−0.056	0.42	0.946	0.894	0.42	2.15
Positive TPOAb (Q2)^c^	0.083	0.10	1.086	0.393	0.90	1.31	0.175	0.26	1.192	0.494	0.72	1.97
Positive TPOAb (Q3)^c^	−0.065	0.15	0.937	0.666	0.69	1.27	0.210	0.40	1.233	0.599	0.56	2.70
Positive TPOAb (Q4)^c^	0.061	0.13	1.063	0.614	0.82	1.38	0.384	0.44	1.469	0.386	0.62	3.50
Age	0.448	0.10	1.565	**<0.001**	1.28	1.91	0.512	0.25	1.669	**0.041**	1.02	2.73
Female^a^	0.222	0.06	1.249	**<0.001**	1.12	1.39	0.301	0.22	1.351	0.178	0.87	2.09
Diabetes (Yes)^b^	−0.067	0.09	0.935	0.434	0.79	1.11	0.098	0.28	1.103	0.724	0.64	1.90

Q, Quartile; ^a^ compared with male; ^b^ compared with non-diabetes; ^c^ compared with negative cases; SE, standard error; PR, prevalence ratio; CI, confidence interval; LL, lower limit; UL, upper limit; OA, osteoarthritis. Significant values are in bold text.

When evaluating the relationship between TPOAb and symptomatic hand OA, the results from a robust Poisson regression revealed that higher levels of TPOAb were associated significantly with a higher prevalence of symptomatic hand OA when no covariates were controlled for, *PR* = 1.182 (95% CI: 1.02, 1.37), *p* = 0.024. When adjusting for age, gender, and diabetes, TPOAb was also found to be significantly related to symptomatic hand OA, *PR* = 1.174 (95% CI: 1.01, 1.37), *p* = 0.039. Specifically, participants with 1 SD increase in TPOAb values were 1.174 times more likely to report symptomatic hand OA (see Table 4, models 1 and 2).

Positive TPOAb was then categorized into quartiles and each positive level was compared with negative TPOAb. Models 3 and 4, in Table 4, demonstrated that no significant differences were found between any quartile level of positive TPOAb and negative TPOAb on the prevalence of hand OA or of symptomatic hand OA (all *p*s > 0.05), regardless of whether covariates were controlled for or not.

#### Associations between anti-thyroglobulin antibody and hand OA

To examine the relationships between TgAb and hand OA/symptomatic hand OA, TgAb was first treated as a continuous variable. In both the unadjusted model and adjusted model (age, gender, and diabetes as covariates), no significant relationship was found between TgAb and hand OA (Table 5, models 1 and 2). Nevertheless, age and gender were found to be significantly related to hand OA. Similar to the TPOAb analysis described above, the results shown in Table 5 (model 2) demonstrated that female were more likely to have hand OA than male, *PR* = 1.253 (95% CI: 1.11, 1.41), *p* < 0.001. Older participants were more likely to have hand OA, *PR* = 1.559 (95% CI: 1.28, 1.90), *p* < 0.001. Additionally, TgAb as a continuous variable was not significantly associated with symptomatic hand OA regardless of whether controlling for covariates or not, although there was a positive trend toward association (*p* = 0.071 and *p* = 0.094, respectively) (Table 5, models 1 and 2).

**Table 5:** Associations between TgAb and hand OA/symptomatic hand OA.

	Hand OA						Symptomatic hand OA					
					95% CI of PR						95% CI of PR	
Models	*β*	SE	PR	*p*	LL	UL	*β*	SE	PR	*p*	LL	UL
Model 1												
TgAb	−0.014	0.05	0.986	0.771	0.90	1.09	0.086	0.05	1.089	0.071	0.99	1.20
Model 2												
TgAb	−0.020	0.06	0.980	0.733	0.88	1.09	0.085	0.05	1.089	0.094	0.99	1.20
Age	0.444	0.10	1.559	**<0.001**	1.28	1.90	0.499	0.28	1.648	0.088	0.96	2.84
Female^a^	0.225	0.06	1.253	**<0.001**	1.11	1.41	0.330	0.19	1.390	**0.038**	0.96	2.00
Diabetes (Yes)^b^	−0.063	0.09	0.939	0.462	0.79	1.11	0.102	0.24	1.108	0.669	0.69	1.77
Model 3												
Positive TgAb (Q1)^c^	−0.070	0.17	0.933	0.674	0.67	1.29	−0.595	0.70	0.552	0.393	0.14	2.16
Positive TgAb (Q2)^c^	−0.043	0.17	0.958	0.800	0.69	1.34	−0.054	0.40	0.947	0.893	0.43	2.08
Positive TgAb (Q3)^c^	0.192	0.18	1.212	0.272	0.86	1.71	−0.454	0.62	0.635	0.464	0.19	2.14
Positive TgAb (Q4)^c^	0.123	0.12	1.131	0.293	0.90	1.42	0.807	0.30	2.242	**0.008**	1.24	4.05
Model 4												
Positive TgAb (Q1)^c^	−0.079	0.16	0.924	0.627	0.67	1.27	−0.618	0.72	0.539	0.389	0.13	2.20
Positive TgAb (Q2)^c^	−0.080	0.16	0.923	0.608	0.68	1.25	−0.113	0.39	0.893	0.772	0.42	1.92
Positive TgAb (Q3)^c^	0.104	0.16	1.110	0.523	0.81	1.53	−0.560	0.61	0.571	0.359	0.17	1.89
Positive TgAb (Q4)^c^	0.031	0.12	1.032	0.816	0.81	1.31	0.715	0.33	2.045	**0.038**	1.07	3.92
Age	0.500	0.10	1.648	**<0.001**	1.34	2.02	0.463	0.25	1.589	0.066	0.97	2.60
Female^a^	0.225	0.06	1.252	**< 0.001**	1.12	1.40	0.333	0.22	1.395	0.159	0.91	2.15
Diabetes (Yes)^b^	−0.067	0.09	0.935	0.472	0.78	1.12	0.126	0.28	1.134	0.651	0.66	1.96

Q, Quartile; ^a^ compared with male; ^b^ compared with non-diabetes; ^c^ compared with negative cases; SE, standard error; PR, prevalence ratio; CI, confidence interval; LL, lower limit; UL, upper limit; OA, osteoarthritis. Significant values are in bold text.

When positive TgAb was categorized into quartiles and each quartile level was compared with negative TgAb, no significant differences were found in the prevalence of hand OA between any levels of positive and negative TgAb (all *p*s > 0.05) in either unadjusted or adjusted models (Table 5, models 3 and 4). However, there was a significant relationship between categorized TgAb and symptomatic hand OA. As shown in Table 5, models 3 and 4, participants with higher levels of TgAb in the fourth quartile (> 16.15 IU/mL) had a significantly higher prevalence of symptomatic hand OA than the participants with negative TgAb in the unadjusted model [*PR* = 2.242, 95% CI (1.24, 4.05), *p* = 0.008] and in the adjusted model [*PR* = 2.045, 95% CI (1.07, 3.92), *p* = 0.038].

**Figure 2:**
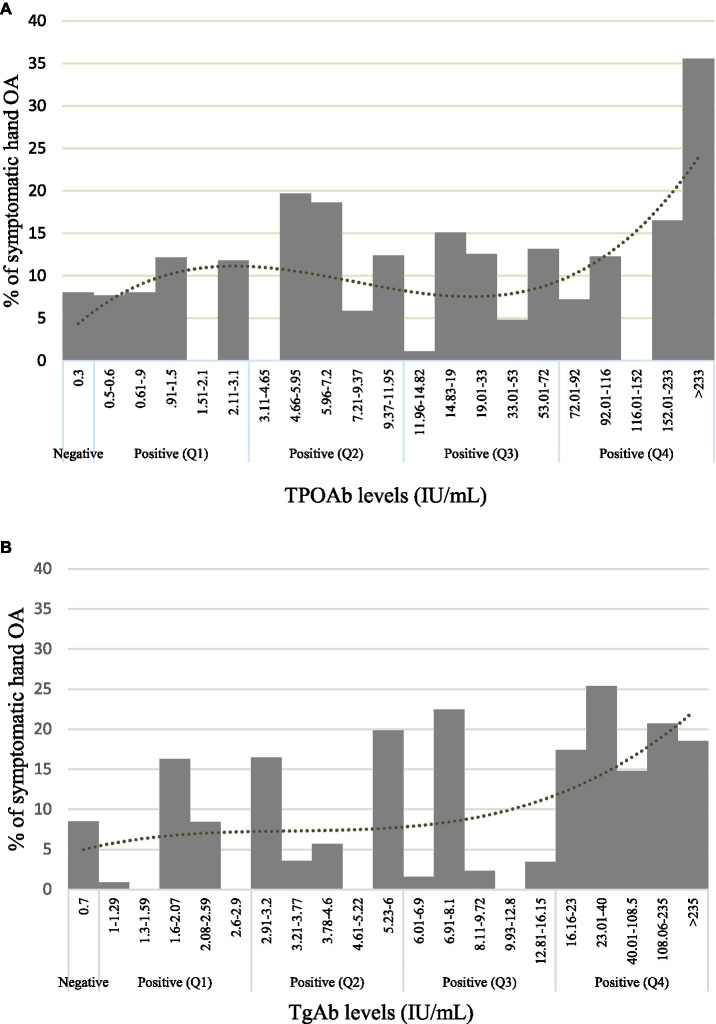
Prevalence of symptomatic hand OA. **(A)** TPOAb levels and **(B)** TgAb levels are separated by negative levels and positive quartiles.

In summary, higher levels of TPOAb were significantly associated with a higher prevalence of symptomatic hand OA. By contrast, with TgAb, a positive trend was shown between TgAb levels and symptomatic hand OA (*p* < 0.10), while a significantly higher prevalence of symptomatic hand OA was identified in the fourth and highest quartile level of TgAb as compared with negative TgAb. The percentage of symptomatic hand OA within the various TPOAb and TgAb levels, displayed in Figures 2A,B, suggests the dose–response relationship between the ATA and symptomatic hand OA. Neither TPOAb nor TgAb was significantly related to hand OA. We performed further analyses comparing each level of positive TPOAb and TgAb by quintiles with negative levels to examine the consistency of any possible dose relationship. The relationships were maintained and the highest quintile level of TgAb (>23.00 IU/mL) (Supplementary Figure S1) was significantly related to a higher prevalence of symptomatic hand OA as compared with negative TgAb in the unadjusted model [*PR* = 2.280, 95% CI (1.16, 4.48), *p* = 0.017] and in the adjusted model (*PR* = 2.111, 95% CI [1.03, 4.34], *p* = 0.042). The relationships were similarly maintained with TPOAb, which was not significant when assessed as a categorical variable by quintiles in both unadjusted and adjusted models (all *ps* > 0.05).

## Discussion

We have demonstrated the association of the ATA, TPOAb, and TgAb as continuous and categorical variables, respectively, with the presence of symptomatic hand OA in a cohort representative of the US population and unselected for the presence of rheumatic disease. The risk of symptomatic hand OA in the TPOAb group was the highest in those with serum concentrations >233 IU/mL, of whom, approximately 35% reported symptomatic hand OA (Figure 2A). A similar trend was seen with the TgAb group, in whom the highest quartile of ≥16 IU/mL had higher levels of symptomatic hand OA. However, the association of TPOAb with symptomatic hand OA did not reach statistical significance when stratified into quartiles, and we speculate that a larger sample size might be required to further explore a dose-dependent association. There was no statistical significance in the associations of either autoantibody with hand OA. The report by Addimanda et al. showing the association of hand OA with AITD in a hospital-based cohort suffered from several limitations, including small subject numbers and potential selection bias (10). Our study demonstrated an association with symptomatic hand OA in the general population but perhaps lacked the sensitivity to detect the same with hand OA given the sample characteristics. As observed in other studies, age and female gender were highly associated with hand OA, and age with symptomatic hand OA (3, 18–20). The age and gender associations were seen with the bony deformities, and the first CMC deformity was also associated with diabetes by participant report in the univariate model, but diabetes was not associated with OA or symptomatic OA in the adjusted models. In our analysis, BMI was not associated with hand OA or symptomatic hand OA. The data suggest that the thyroid autoantibodies are associated more closely with chronic hand pain than with bony deformities (Supplementary Figure S2). We have been able to confirm the association with hand pain, in particular in a separate study using the NHANES III dataset (21). Our findings suggest that approximately 10–20% of the adult population ≥ 60 years with AITD may be at risk for symptomatic hand OA, and that risk factor would have to be considered in the clinical presentation of hand OA. This would add to other disease associations, including non-rheumatic sequelae and rheumatic manifestations of AITD, including fibromyalgia syndrome, spinal OA, and chondrocalcinosis (17, 22–24).

We did not measure associations of our outcome measures with the TSH receptor antibody, which was not assayed in NHANES III, is less important in case definitions, and has not been shown to associate with the musculoskeletal manifestations of AITD (11, 25). Our study is the first to suggest that there is a clinical phenotype associated with TgAb at the highest serum concentrations by way of symptomatic hand OA. This was an unexpected finding since, unlike the TPOAb, the TgAb does not fix complement and has not been implicated in the pathogenesis of hypothyroidism or hyperthyroidism (26). However, it may be that higher concentrations of TgAb are associated more with established or severe AITD, which may be more highly associated with OA. We did not see a robust correlation of the TPOAb with the TgAb but cannot completely exclude collinearity as a cause of the finding. There was no association of hand OA or symptomatic hand OA with TSH levels (Supplementary Figure S3).

Proposed mechanisms for the pathogenesis of hand OA include localized damage to cartilage from joint stress or injury, resulting in local inflammatory changes (27, 28). Traditional risk factors, such as increased BMI, produce *in situ* mechanotransduction forces that cause local inflammation but have been proposed as risk factors for more systemic inflammation in part via the metabolic syndrome, adipokine release, and activation of serum acute phase reactants. This provides some explanation for how an indirect effect of BMI could cause systemic inflammation and hand OA at a distance from the inciting influence (28, 29). We did observe a positive relationship between TPOAb and TgAb (r = 0.257) between the serum acute phase reactant CRP and TPOAb (r = 0.123) and between CRP and TgAb (r = 0.046), but the associations were weak. We did not examine the relationships at the highest concentrations of the ATA because of the small numbers involved. Therefore, our data using this retrospective cohort did not support a clear role for inflammation explaining the AITD and symptomatic hand OA association and did not show a role for BMI in hand OA (Supplementary Figure S4).

Our derivation of clinical hand OA from NHANES III, which emphasized the decision tree classification of the ACR, compared very well with the findings of Dillon et al. (14). However, our overall findings require corroboration in a prospective cohort study using more clinically comprehensive and sensitive methods of diagnosing hand OA.

The limitations of the study include the retrospective nature of the study cohort and the difficulty in determining causation. The use of a population-based sample to study any disease association could suffer from sampling bias and type II error if the frequency of the association is hampered by the sample size (30). We therefore cannot completely exclude an association between the ATA and the hand deformities in OA, particularly at the higher concentrations of the ATA, especially since the trends except for the first CMC deformity favored participants with TPOAb despite their lack of statistical significance (Supplementary Figure S2). The use of the antibodies as a surrogate for thyroid autoimmunity, though accepted in epidemiological studies, may not capture all patients with AITD using more extensive methods of classification, including thyroid function tests, physical thyroid examination, thyroid ultrasonography, and fine needle aspiration of the thyroid gland (31). Furthermore, NHANES III used a highly sensitive radioimmunological assay for measuring the ATA that allowed clear separation of normal and abnormal values, which may not be possible using modern, less sensitive ELISA-based techniques and may thus prevent fair comparison between studies. Finally, the results cannot be generalized to a younger age group of <60 years since NHANES III did not capture OA data on that age group.

In summary, we have investigated the associations of TPOAb and TgAb with clinical hand OA in a sample representative of older individuals in the US population. The findings, which suggest an association with symptomatic hand OA in an unselected population aged 60 years and over, add to growing evidence that AITD, the commonest autoimmune disease, has a clinical expression by way of rheumatologic sequelae that includes OA and chronic pain syndromes (11). Further studies would require closer investigation using prospective cohorts of AITD patients with documentation of clinical findings, markers of inflammation, and other serologies to explore causation, probable mechanisms of injury, and any possible dose dependency between the ATA and the rheumatic phenomena (24).

## Data Availability

Publicly available datasets were analyzed in this study. This data can be found at: https://wwwn.cdc.gov/nchs/nhanes/nhanes3/default.aspx.
