# Supplementary material for: Autoimmune thyroid disease modifies the clinical expression of hand osteoarthritis in older people: a third National Health and nutrition examination survey study
Source: Front Med (Lausanne). 2024 Aug 20;11:1445188. doi: 10.3389/fmed.2024.1445188 (PMC11368861; doi:10.3389/fmed.2024.1445188)
Supplement: Supplementary file 1 [file Data_Sheet_1.docx]

**APPENDIX:**

**Supplementary Material**

Supplementary data analysis items and rationale.

Supplement Table 1. STROBE Checklist.

Supplement Figure 1. Analysis of prevalence of symptomatic hand OA by anti-thyroid antibody levels measured in quintiles.

Supplement Figure 2. Analysis of the data suggested that the thyroid autoantibodies associate more closely with chronic hand pain than with the bony deformities in examining the association with the various components of hand OA.

Supplement Figure 3. Examination of the association of thyroid function represented by TSH levels with hand OA and symptomatic hand OA failed to reveal any significant relationships.

Supplement Figure 4. Analysis of the relationship between BMI and hand OA/symptomatic hand OA failed to reveal an association in the NHANES III data set.

Supplement Legends

Supplement Table 1. STROBE Checklist.

Supplement Figure 1. Prevalence of symptomatic hand OA by A). TPOAb levels and B). TgAb levels separated by negative level and positive quintiles.

Supplement Figure 2. Percentages of negative and positive TPOAb/TgAb in each group of hand pain, Heberden’s nodes, Bouchard’s nodes, and first CMC deformity. A greater proportion of participants with hand pain had positive TPOAb than those without hand pain (*X*^2^ = 5.06, *p* = 0.025, *N* = 2423). There was no significant relationship between TPOAb and Heberden's nodes (*X*^2^ = 1.94, *p* = 0.163, *N* = 2354), Bouchard's nodes (*X*^2^ = 3.40, *p* = 0.065, *N* = 2354), and first CMC deformity (*X*^2^ = 0.01, *p* = 0.929, *N* = 2350). A greater proportion of participants with hand pain (21.2%) had positive TgAb than those without hand pain(16.8%) (*X*^2^ = 4.18, *p* = 0.041, *N* = 2423). There was no significant relationship between TgAb and Heberden's nodes (*X*^2^ = 2.11, *p* = 0.146, *N* = 2354), Bouchard's nodes (*X*^2^ = 2.00, *p* = 0.157, *N* = 2354), and first CMC deformity (*X*^2^ = 0.21, *p* = 0.649, *N* = 2350).

Supplement Figure 3. Percentages of TSH levels in each group in Hand OA and in symptomatic hand OA. Rao-Scott *X*^2^ = 1.91, *p* = 0.385 for the relationship between TSH and hand OA; Rao-Scott *X*^2^ = 0.57, *p* = 0.751 for the relationship between TSH and symptomatic hand OA.

Supplement Figure 4. Mean and SD of BMI in each group of hand OA and symptomatic hand OA. Participants with hand OA appear to have lower BMI than those without hand OA (*t* = 3.68, *p* = 0.001), but the difference did not reach clinical significance (M_BMI_ = 26.66 with hand OA vs. M_BMI_ = 27.58 without hand OA). No significant difference of BMI was identified between participants with symptomatic hand OA and participants without symptomatic hand OA (*t* = 0.69, *p* = 0.497).

Supplement Figure 1

Supplement Figure 2

Supplement Figure 3

Supplement Figure 4

Supplement Table 1. STROBE Statement—checklist of items that should be included in reports of observational studies

|  | Item No. | Recommendation | Page  No. | Relevant text from manuscript |
| --- | --- | --- | --- | --- |
| **Title and abstract** | 1 | (*a*) Indicate the study’s design with a commonly used term in the title or the abstract | 1,6 | NHANES III stated in title |
|  |  | (*b*) Provide in the abstract an informative and balanced summary of what was done and what was found | 2 |  |
| Introduction | | | |  |
| Background/rationale | 2 | Explain the scientific background and rationale for the investigation being reported | 4-5 |  |
| Objectives | 3 | State specific objectives, including any prespecified hypotheses | 5 |  |
| Methods | | | |  |
| Study design | 4 | Present key elements of study design early in the paper | 5-9 |  |
| Setting | 5 | Describe the setting, locations, and relevant dates, including periods of recruitment, exposure, follow-up, and data collection | 5-9 |  |
| Participants | 6 | (*a*) *Cohort study*—Give the eligibility criteria, and the sources and methods of selection of participants. Describe methods of follow-up  *Case-control study*—Give the eligibility criteria, and the sources and methods of case ascertainment and control selection. Give the rationale for the choice of cases and controls  *Cross-sectional study*—Give the eligibility criteria, and the sources and methods of selection of participants | 5-9 |  |
|  |  | (*b*) *Cohort study*—For matched studies, give matching criteria and number of exposed and unexposed  *Case-control study*—For matched studies, give matching criteria and the number of controls per case |  |  |
| Variables | 7 | Clearly define all outcomes, exposures, predictors, potential confounders, and effect modifiers. Give diagnostic criteria, if applicable | 8 |  |
| Data sources/ measurement | 8* | For each variable of interest, give sources of data and details of methods of assessment (measurement). Describe comparability of assessment methods if there is more than one group | 7 |  |
| Bias | 9 | Describe any efforts to address potential sources of bias | 9 |  |
| Study size | 10 | Explain how the study size was arrived at | 5-6 | Full data source was used |

Continued on next page

| Quantitative variables | 11 | Explain how quantitative variables were handled in the analyses. If applicable, describe which groupings were chosen and why | 9 |  |
| --- | --- | --- | --- | --- |
| Statistical methods | 12 | (*a*) Describe all statistical methods, including those used to control for confounding | 9 |  |
|  |  | (*b*) Describe any methods used to examine subgroups and interactions | 9 |  |
|  |  | (*c*) Explain how missing data were addressed | 9 |  |
|  |  | (*d*) *Cohort study*—If applicable, explain how loss to follow-up was addressed  *Case-control study*—If applicable, explain how matching of cases and controls was addressed  *Cross-sectional study*—If applicable, describe analytical methods taking account of sampling strategy | 9 |  |
|  |  | (*e*) Describe any sensitivity analyses |  |  |
| Results | | | | |
| Participants | 13* | (a) Report numbers of individuals at each stage of study—eg numbers potentially eligible, examined for eligibility, confirmed eligible, included in the study, completing follow-up, and analysed | 10 | Figure 1 |
|  |  | (b) Give reasons for non-participation at each stage | 10 | Figure 1 |
|  |  | (c) Consider use of a flow diagram | 10 | Figure 1 |
| Descriptive data | 14* | (a) Give characteristics of study participants (eg demographic, clinical, social) and information on exposures and potential confounders | 10-12 | Table 2 |
|  |  | (b) Indicate number of participants with missing data for each variable of interest | 10-12 | Figure 1 |
|  |  | (c) *Cohort study*—Summarise follow-up time (eg, average and total amount) |  |  |
| Outcome data | 15* | *Cohort study*—Report numbers of outcome events or summary measures over time |  |  |
|  |  | *Case-control study—*Report numbers in each exposure category, or summary measures of exposure |  |  |
|  |  | *Cross-sectional study—*Report numbers of outcome events or summary measures | 10-12 | Result tables |
| Main results | 16 | (*a*) Give unadjusted estimates and, if applicable, confounder-adjusted estimates and their precision (eg, 95% confidence interval). Make clear which confounders were adjusted for and why they were included | 10-12 |  |
|  |  | (*b*) Report category boundaries when continuous variables were categorized | 10-12 |  |
|  |  | (*c*) If relevant, consider translating estimates of relative risk into absolute risk for a meaningful time period |  |  |

Continued on next page

| Other analyses | 17 | Report other analyses done—eg analyses of subgroups and interactions, and sensitivity analyses | 10-12 |  |
| --- | --- | --- | --- | --- |
| Discussion | | | | |
| Key results | 18 | Summarise key results with reference to study objectives | 12-13 |  |
| Limitations | 19 | Discuss limitations of the study, taking into account sources of potential bias or imprecision. Discuss both direction and magnitude of any potential bias | 15 |  |
| Interpretation | 20 | Give a cautious overall interpretation of results considering objectives, limitations, multiplicity of analyses, results from similar studies, and other relevant evidence | 12-16 |  |
| Generalisability | 21 | Discuss the generalisability (external validity) of the study results | 15-16 |  |
| Other information | |  | | |
| Funding | 22 | Give the source of funding and the role of the funders for the present study and, if applicable, for the original study on which the present article is based |  | No external sources of funding |

*Give information separately for cases and controls in case-control studies and, if applicable, for exposed and unexposed groups in cohort and cross-sectional studies.

**Note:** An Explanation and Elaboration article discusses each checklist item and gives methodological background and published examples of transparent reporting. The STROBE checklist is best used in conjunction with this article (freely available on the Web sites of PLoS Medicine at http://www.plosmedicine.org/, Annals of Internal Medicine at http://www.annals.org/, and Epidemiology at http://www.epidem.com/). Information on the STROBE Initiative is available at www.strobe-statement.org.
